# Vital signs during complication-free recovery after major surgery: a retrospective analysis

**DOI:** 10.1007/s10877-026-01471-7

**Published:** 2026-07-11

**Authors:** Casper H. Guldager, Jesper Mølgaard, Helle K. Hansen, Christian S. Meyhoff, Eske K. Aasvang

**Affiliations:** 1 Department of Anesthesiology, Center for Cancer and Organ Diseases, Rigshospitalet, University of Copenhagen, Blegdamsvej 9, Copenhagen, 2100 Denmark; 2 Department of Anaesthesia and Intensive Care, Copenhagen University Hospital - Bispebjerg and Frederiksberg, Bispebjerg Bakke 23, Copenhagen, 2400 Denmark; 3 Department of Clinical Medicine, University of Copenhagen, Blegdamsvej 3, Copenhagen, 2100 Denmark

**Keywords:** Vital signs, Major surgery, Reference values, Continuous monitoring

## Abstract

Continuous vital sign monitoring (CVSM) is superior to intermittent manual vital sign assessment in detecting deviations, but alert thresholds have limited evidence, as a strong definition of physiologically normal variations are lacking. Consequences of too few alerts are missed detections of oncoming complications, and too many alerts result in over-alerting and over-treatment. This study aimed to describe duration and deviation of vital signs in postoperative patients without complications when monitored continuously. Adult patients admitted to the general ward after major surgery were included. Continuous wireless vital sign monitoring (CVSM) was performed during the early postoperative period, with data collection blinded to ward nurses. For peripheral oxygen saturation (SpO₂), systolic blood pressure, heart rate, and respiratory rate, the longest consecutive durations outside predefined thresholds were identified. The primary outcome was the longest consecutive duration of SpO₂ < 88%. A total of 600 patients were analyzed and of these, 277 patients had no complications of any kind. The longest sustained episodes with SpO_2_ < 88% were median 9 min [IQR 2–31 min]. The maximum length of other vital sign sustained deviations had the following median duration in minutes: SpO_2_ < 92%: 58[6 -129], HR > 110/min: 3 [0–13], HR < 50/min: 0 [0–1], RF > 24/min: 1 [0–4] and RF < 11/min: 4 [2–12]. *Sustained* episodes of severe desaturations, tachycardia and tachypnea were short, but moderate desaturations frequent. These findings indicate that vital signs in complication free patients should be carefully analyzed when monitored continuously after major surgery.

**Supplementary Information:**

The online version contains supplementary material available at https://doi.org/10.1007/s10877-026-01471-7.

## Introduction

Assessing vital signs is an integrated daily part of postoperative patient monitoring. However, despite numerous publications alert thresholds are not always evidence based, and often based on intermittent, nurse led data collection [1–4]. One foundational understanding is that that deviations from normal physiology must by default be harmful and should be corrected.

As a result, continuous vital sign monitoring produces a substantial number of irrelevant alerts both in the OR, ICU and PACU [5, 6], which to some extent is handled due to the high nurse: patient ratio, yet still with the inherent risk for alert fatigue [7, 8] at the general ward, this is, however, not the case.

Substantial efforts to implement ICU/PACU grade continuous vital sign monitoring (CVSM) technology are currently aimed at the general ward. Fueled by the recognition that the risk for postoperative complications does not end after PACU-discharge but occurs in two-figured percentages of cases [9–13] in the general ward. So far, the clinical standard with track and trigger systems for detecting deteriorating patients in hospital wards such as the National Early Warning Score (NEWS) [14] have failed to reduce morbidity and mortality. Due to the manual and intermittent NEWS methodology there is an inherent risk of long and unobserved patient deterioration potentially may allow for deterioration and failure to rescue [3, 15, 16]. Additionally, the thresholds of the NEWS systems, are largely based on expert opinions as there is a paucity of what constitutes normal or dangerous vital sign deviations for a specific patient [1–3]. CVSM can detect deviating vital signs at a much higher rate than track and trigger systems such as the EWS [11, 17–19] with emerging evidence for improved patient outcomes [20, 21]. Even so, using CVSM systems where thresholds and durations are not properly evidence based, or extrapolated from intermittent thresholds risks causing either too many alerts - resulting in alert fatigue and overtreatment – or missing the predictors of complications.

We aimed to describe the duration of vital sign deviations in postoperative patients without complications, primarily peripheral oxygen saturation SpO_2_ < 88%.

We hypothesized that the median duration of SpO_2_ < 88% would be short with a median of 15 min, and other deviations in vital signs including, heart rate (HR), peripheral oxygen saturation (SpO_2_), respiration rate (RR), and systolic blood pressure (SBP), would be of shorter durations following increasing deviation severity.

## Methods

This was a retrospective analysis of patients included in two prospective studies “WARD Surgery Observational [22] [clinicaltrials.gov: NCT03491137]” and “WARD Surgery RCT [21] [clinicaltrials.gov : NCT04640415]”, of continuous vital sign monitoring at the general surgical hospital ward after discharge from the PACU. Both studies were approved by ethics committees [H-20034555] and [H-17033535] and data handling authorities, and patients were included after verbal and written information about the studies.

Patients were assessed for inclusion eligibility age ≥ 50 years and elective major surgery (abdominal, urologic, orthopedic, or vascular procedures), with an estimated surgical duration ≥ 2 h, and a minimum expected two days hospital stay. Study exclusion criteria were severe cognitive impairment assessed by the Mini-Mental State Examination (MMSE), or inability to cooperate in wearing the wireless monitoring equipment. Furthermore, the intervention population from the randomized clinical trial was excluded as staff were given 24/7 information on the intervention groups vital signs and interventions was expected to be affected by interventions. Device producer exclusion criteria were implanted cardioverter defibrillator or pacemaker and allergy to study devices.

Patient care followed recommendations from the Enhanced Recovery After Surgery [23] society.

Adverse events were defined according to the International Conference on Harmonization-Good Clinical Practice-guideline [24] as any unexpected and unfavorable event occurring during a medical trial, regardless of that event is considered related to the ongoing study. Complications were identified using a predefined outcome manual based upon internationally agreed diagnosis criteria. Patients with chart notes of any complications during the first 30 postoperative days were used to as a comparator group. Demographic data on age, sex, BMI, American Society of Anesthesiology (ASA) class, surgical procedure etc. were collected (see Table 1).

**Table 1:** Demographic data

	Overall	No complication	Any complication
*n*	600	277	323
AGE, median [IQR]	70.0 [66.0,75.0]	70.0 [65.0,74.0]	71.0 [66.0,75.0]
Men, n (%)	376 (62.7)	161 (58.1)	215 (66.6)
Height / Cm, mean (SD)	173.0 (9.3)	172.6 (9.1)	173.2 (9.6)
Weight / Kg, mean (SD)	78.6 (16.9)	77.4 (15.5)	79.6 (17.9)
BMI / kg/m^2^, mean (SD)	26.2 (4.7)	25.9 (4.5)	26.4 (4.9)
Smoking Status, n (%)			
Never	186 (31.0)	94 (33.9)	92 (28.5)
Former smoker	328 (54.7)	138 (49.8)	190 (58.8)
Current smoker	84 (14.0)	43 (15.5)	41 (12.7)
For smokers: Smoking Pack years, mean (SD)	32.2 (24.2)	30.5 (22.2)	33.5 (25.6)
Alcohol, n (%)			
None	116 (19.3)	46 (16.6)	70 (21.7)
Not more than MADI	368 (61.3)	177 (63.9)	191 (59.1)
More than MADI	116 (19.3)	54 (19.5)	62 (19.2)
ASA score, n (%)			
1	21 (3.5)	15 (5.4)	6 (1.9)
2	319 (53.2)	171 (61.7)	148 (45.8)
3	254 (42.3)	88 (31.8)	166 (51.4)
4	6 (1.0)	3 (1.1)	3 (0.9)
Preexisting AFLI, n (%)	83 (13.8)	32 (11.6)	51 (15.8)
CCI SCORE, n (%)			
0–2	149 (24.8)	82 (29.6)	67 (20.7)
3	192 (32.0)	88 (31.8)	104 (32.2)
4	127 (21.2)	48 (17.3)	79 (24.5)
5	51 (8.5)	21 (7.6)	30 (9.3)
6+	81 (13.5)	38 (13.7)	43 (13.3)
Procedure type			
Bowel surgery	167 (27.8)	113 (40.8)	54 (16.7)
Pancreatic surgery	170 (28.3)	58 (20.9)	112 (34.7)
Esophageal surgery	77 (12.8)	12 (4.3)	65 (20.1)
Rectal surgery	45 (7.5)	23 (8.3)	22 (6.8)
Gastric and duodenal surgery	42 (7.0)	24 (8.7)	18 (5.6)
Abdominal wall and peritoneal surgery	30 (5.0)	15 (5.4)	15 (4.6)
Biliary surgery	9 (1.5)	5 (1.8)	4 (1.2)
Vascular surgery	25 (4.2)	12 (4.3)	13 (4.0)
Other major non-cardiac surgery	35 (5.8)	15 (5.4)	20 (6.2)
Surgical duration / hours, median [IQR]	4.4 [3.2,5.5]	3.9 [3.1,5.0]	5.0 [3.6,5.8]

Demographic data. Results are given in mean with (Standard deviation) or median with [Interquartile range]

Abbreviations: IQR: interquartile range. Cm: Centimeters, kg: kilograms, MADI; Maximum Advised Daily Intake, ASA (American Society of Anesthesiologists)

### Outcomes

The primary outcome was the maximum sustained duration of SpO_2_ < 88% during any day of observation.

Secondary outcomes were maximum sustained durations of episodes with deviating vital signs outside predefined thresholds of $\:{\mathrm{S}\mathrm{p}\mathrm{O}}_{2}$, HR, RF and SBP (Table 2). These vital sign thresholds were adopted and expanded from NEWS and previous publications [14, 22].

**Table 2:** Maximum duration of vital sign deviations

	No Complications		Any Complications	
	Mean (SD)	Median [IQR]	Mean (SD)	Median [IQR]
Respiratory				
SpO2 < 92%	89.5 (109.6)	58.0 [6.0–129.0]	74.6 (96.8)	40.0 [4.0–102.0]
SpO2 < 88%	27.0 (46.0)	9.0 [2.0–31.0]	33.6 (65.0)	9.0 [2.0–32.0]
SpO2 < 85%	11.2 (29.0)	3.0 [1.0–9.0]	13.4 (37.2)	3.0 [1.0–9.0]
SpO2 < 80%	3.3 (10.6)	1.0 [0.0–3.0]	4.1 (17.0)	1.0 [0.0–2.0]
Respiration Rate < 5 brpm	0.2 (0.7)	0.0 [0.0–0.0]	0.2 (0.7)	0.0 [0.0–0.0]
Respiration Rate < 11 brpm	15.6 (40.4)	4.0 [2.0–12.0]	9.6 (20.7)	3.0 [1.0–8.0]
Respiration Rate > 24 brpm	5.6 (13.5)	1.0 [0.0–4.0]	5.1 (13.6)	1.0 [0.0–4.0]
Respiration Rate > 35 brpm	0.5 (3.3)	0.0 [0.0–0.0]	0.2 (1.2)	0.0 [0.0–0.0]
Circulatory				
Heart Rate < 30	0.2 (1.2)	0.0 [0.0–0.0]	0.1 (0.7)	0.0 [0.0–0.0]
Heart Rate < 50	6.2 (22.9)	0.0 [0.0–1.0]	3.9 (20.7)	0.0 [0.0–1.0]
Heart Rate > 111	14.9 (35.2)	3.0 [0.0–13.0]	24.7 (81.3)	1.0 [0.0–10.5]
Heart Rate > 131	3.9 (14.3)	0.0 [0.0–1.0]	3.4 (18.3)	0.0 [0.0–0.0]
Systolic Blood Pressure < 70	0.1 (0.9)	0.0 [0.0–0.0]	0.2 (1.3)	0.0 [0.0–0.0]
Systolic Blood Pressure < 90	2.8 (6.5)	0.0 [0.0–2.1]	5.4 (12.4)	0.0 [0.0–4.7]
Systolic Blood Pressure > 180	3.0 (9.6)	0.0 [0.0–2.1]	1.9 (6.0)	0.0 [0.0–0.0]
Systolic Blood Pressure > 220	0.4 (3.1)	0.0 [0.0–0.0]	0.2 (1.2)	0.0 [0.0–0.0]

Table describing statistical characteristics of maximum duration of vital sign deviations after major surgery, recorded continuous wireless monitoring for up to 3 postoperative days. Systolic blood pressure values are described in percent of all measurements

SpO_2_ = peripheral oxygen saturation, brpm = breaths per minute

### Data collection

The WARD-project CVSM setup has previously been described [22], but overall consisted of $\:{\mathrm{S}\mathrm{p}\mathrm{O}}_{2}$, HR, RR and SBP measured for up to five days post-operatively upon patient arrival at the general ward using wireless vital sign monitors. In the current study we only included data from the first 3 days due to loss of patients because of hospital discharge. Vital sign data from patients with complications were censored at first moment of clinical confirmation (e.g. confirmation by imaging or blood test). All continuously recorded data were blinded to patient-care staff, which relied on traditional NEWS protocols.

Data on complications were extracted manually from patient medical records by physicians using a predefined protocol including international definitions of complications, ensuring standardized assessment.

Devices and parameters: HR and RR were measured continuously using a single-lead ECG patch (Lifetouch, Isansys, Oxfordshire, UK). Peripheral oxygen saturation (SpO_2_) was measured continuously by a finger pulse-oximeter (Nonin Wristox 3150, Nonin Medical Inc, MN 55441, USA) measuring (SpO_2_) every minute. Blood pressure was measured every 30 min during daytime and every 60 min during nighttime using a cuff-based blood pressure monitor (TM-2441, A&D Medical, Tokyo, Japan). All devices were clinically validated [25, 26]. Patients were allowed to discontinue use of any device in case of discomfort. Data was transformed to 1-minute averages, and artifacts were removed. Thus, each episode consists of subsequent 1-minute averages of a deviation in vital signs, except for SBP due to the 30- and 60-minute intervals. As the SBP was not continuously monitored, deviations were described as a percentage of all SBP measurements.

### Analysis

All eligible patients were included in the analysis; thus, no formal power calculation was performed although included in both original studies. To avoid overinflating results based on short monitoring durations, only patients with at least 8 h of data were included. Analyses were based upon the actual recorded data since compliance was not 100% for all devices similar to other continuous vital sign studies [27, 28], resulting in the durations potentially representing a minimum. To assess the correct statistical method for data description, data distribution using mean and standard deviation was examined for raw and log-transformed (log_10_) data using skewness and kurtosis with the Shapiro-Wilks test for normality to determine goodness-of-fit. Based upon this analysis, data were described by medians with 25th and 75th percentiles and furthermore visually by boxplots. This was done for the true continuous data derived from the ECG and saturation-monitor. For the SBP-data the percentage of deviating measurements were calculated.

Primary analysis: For each vital sign parameter and predefined thresholds, the full monitoring period was used.

Additionally, specific vital sign parameter and predefined thresholds were calculated on a per-day basis.

Exploratory analyses included stratification of specific vital sign parameter and predefined thresholds, by time of day (07:00–23:00 vs. 23:00–07:00).

Day 1 was the first day of monitoring. In some instances, monitoring began on the day of the surgery, in other cases monitoring began on the day after the surgery for logistical reasons such as overnight post-anesthesia care unit stay.

Specifically for the oxygen saturation threshold SpO_2_ < 92% patients diagnosed with Chronic Obstructive Pulmonary Disease (COPD) and/or that had a body mass index (BMI) ≥ 40 kg/m2, were excluded because a lower threshold for SpO_2_ is accepted in these patient groups [29].

Statistical analysis and data handling was made in Python v. 3.13.7 using pandas v. 2.3.3.

## Results

Patients were enrolled between February 2018 and July 2020 but halted between March 9, 2020—May15, 2020, in the observational study, and between January 2021 and October 2021 for the RCT due to the Covid 19 pandemic.

**Fig. 1:**
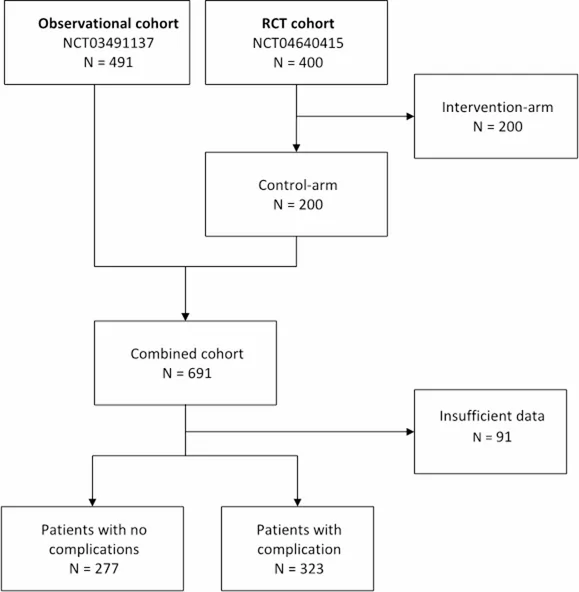
Consort diagram.Figure describing the patient selection process

A total of *n* = 691 unique patient cases were evaluated for eligibility. Missing data occurred in 91 patients, leaving a total of 600 patients. Of these patients, 277 did not have any 30-day complications of any kind, and 323 patients had some measure of complication during 30-days (Fig. 1). Of the all patients eligible for analysis, all (100%) accepted to wear the single lead ECG patch and all (100%) the pulse oximeter whereas (90%) were monitored with the BP-device. Exclusion from the SpO_2_ < 92% analysis was the case for *n* = 78 patients due to COPD and/or a BMI > 40 kg/m2 .

**Fig. 2:**
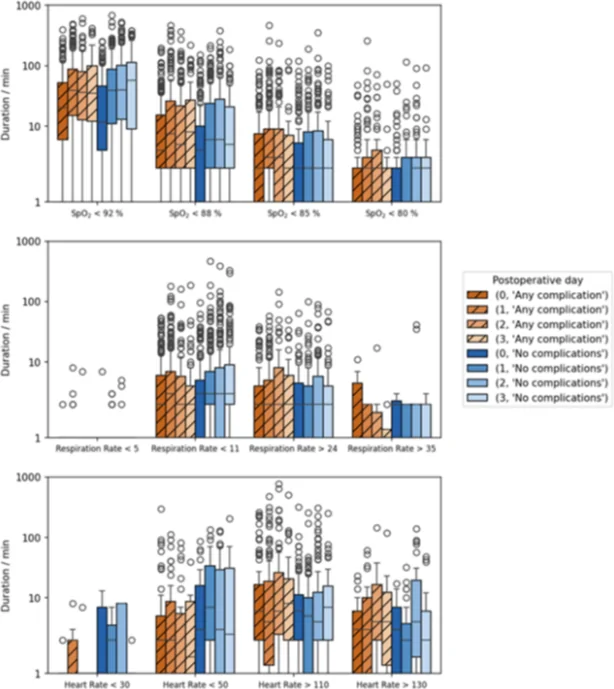
Maximum duration of sustained vital sign episodes in postoperative major surgery patients. Split by each postoperative day. Longest episodes of vital sign deviations outside selected thresholds. Orange / Blue colour and hashing indicate patients with complications or no complications. Split by each postoperative day. Boxes represent 25th to 75th percentile, the median is represented by the middle bar with outliers represented by circles SpO_2_ = Peripheral oxygen saturation

Most patients were men (62.7%) with a median age of 70 years [IQR 66–75], and *n* = 90% had undergone abdominal surgery. The majority had an ASA score of either II or III *n* = 573 (96%). Patient demographics are illustrated in Table 1.

### Primary outcome

The longest episodes with $\:{\mathrm{S}\mathrm{p}\mathrm{O}}_{2}$ < 88% lasted a median 9 min [IQR 2–31] with the 5’th and 95’th percentiles being 0 and 115 min respectively (Table 2; Fig. 2 and supplemental table 1a). For the population with complications, median duration was 9 [IQR 3–32] minutes.

### Secondary outcomes

Overall, there were an inverse relation between severity and durations for all vital sign parameters. Details for all vital sign parameters stratified for each of the first three postoperative days are described in Table 2; Fig. 2 and further detailed in online supplementary table 1a and 1b.

### Oxygen saturation

The longest episodes with Sp$\:{\mathrm{O}}_{2}$ < 92% lasted a median of 58 [IQR 6–129] minutes with 5’th and 95’th percentiles of 0 and 304 min respectively. The longest episodes of the most severe deviation of Sp$\:{\mathrm{O}}_{2}$ < 80% lasted a median of 1 [IQR 0–3] minutes with 5’th and 95’th percentile of 0 and 14 min respectively (Table 2; Fig. 2).

### Respiration rate

The longest episodes RR > 24/minute lasted a median 1 [IQR 0–4] minutes with 5’th and 95’th percentiles of 0 and 23 min respectively. Bradypnea episodes of RR < 11/minute lasted a median of 4 [IQR 2–12] minutes with 5’th and 95’th percentile of 0 and 67 min respectively (Table 2; Fig. 2).

### Heart rate

The longest episodes with HR > 111 bpm lasted a median 3 [IQR 0–13] minutes with 5’th and 95’th percentiles of 0 and 70 min respectively. Bradycardia episodes of HR < 50 bpm lasted a median of 0 [IQR: 0–1] minutes with 5’th and 95’th percentile of 0 and 42 min respectively (Table 2; Fig. 2).

### Systolic blood pressure

The median percent of episodes with hypotension SBP < 90 mmHg occurred a median of 0 [IQR: 0–2] % of measurements, with 5’th and 95’th percentile of 0 and 17% of all measurements respectively. Hypertension episodes of SBP > 180 mmHg occurred a median of 0 [IQR 0–2] % of measurements, with 5’th and 95’th percentile of 0 and 13% of all measurements (Table 2).

### Day- and night-analyses

Daytime analyses of the longest episodes of $\:\mathrm{S}\mathrm{p}{\mathrm{O}}_{2}$ < 88% found a median duration of 2 min on day 1 and a median duration of 1 min on day 2 and 3. Whereas the longest episodes during nighttime lasted a median 0 min on day 2 and a median 0 min on day 3.

## Discussion

This observational study found the median duration of desaturation below 88% was 9 min and 5% of patients had durations exceeding 1115 min in patients without complications after major surgery, thus not supporting our hypothesis. Deviations from widely agreed upon thresholds for normal physiology was common across the various vital sign parameters, with a common pattern of an inverse relation between deviation severity and duration, although with parameter specific patterns such as frequent moderate desaturations. However, only short durations of tachy -cardia and -pnea was observed with a median HR > 111 bpm of 3 min, and tachypnea > 24 brpm of 1 min.

According to NEWS guidelines a single manually measured episode of SpO_2_ < 88% would give a score of 3 thus leading to action in form of a rapid response team even if it is the only deviation. Our data indicate that although this deviation is frequent, the median duration was only 9 min and only 25% of cases lasted longer than 31 min, and more severe deviations were much shorter. Although the results potentially can be used to define abnormal durations for specific thresholds, caution should be taken as to whether shorter durations are safe for individual patients.

A counterintuitive observation was that uncomplicated patients had longer durations for some deviations, notably SpO2 < 92% (median 58 vs. 40 min) and RR < 11 (median 4 vs. 3 min).

In analyzing the episodes of deviating heart rate, we found that severe episodes of tachycardia (HR > 130 bpm) were very uncommon with only 5% of patients having episodes lasting > 20 min, suggesting they should not be accepted or disregarded. In contrast, previous publications have demonstrated episodes of HR > 130 bpm lasting more than 30 min to occur in 7% of patients with SAEs [22].

The current findings can be considered a step into what should have been the foundation for alert systems before the introduction into the clinic. Thus, CVSM systems are undergoing rapid development with clinical implementation taking place at an increasing pace [7], based on the assumption that vital sign deviations can be a predictor of clinically relevant complications [1, 3, 15, 30]. How to use the findings is arguable, including if alerts should be based upon deviations from the 50th, 75th or 99th percentile for instance, with implications for sensitivity and specificity for detecting complications. Thus, we might accept a low specificity for cardiac arrest to improve sensitivity, whereas an alert for eczema should be the opposite.

CVSM has been building evidence for large impact on patient safety including large case-control series and lately also randomized controlled trials [20, 21]. This is made possible by recent technological advances in wearable sensor technology have made it possible to evaluate and analyze CVSM in patients admitted to a regular surgical ward, where observation by medical staff is less intense than in Intensive Care Units (ICUs) or intermediary wards [8, 12]. However, reducing alert frequency is particularly important in the ward setting, due to the lower staffing, but there is a paucity of data on what constitutes normal postoperative vital signs and how these may vary throughout the post operative recovery period. The deviations in vital signs are potentially multifactorial as surgical stress may induce changes in respiration and circulation as part of the normal recovery and healing process [23], much like the post-exercise situation. As shown in supplementary Table 2 there are large variations in deviations for different procedure types. This also necessitates adding contextual knowledge to interpretation of vital sign deviations.

Furthermore, the crucial mobility may increase the respiratory and circulatory work, especially in cases of pain, anemia etc. and the well-described circadian rhythm also results in fluctuations in vital signs [31, 32]. Therefore, what constitutes normal postoperative vital signs [33] must be defined to discriminate against pathological conditions and reduce the burden of false positive alerts and alert fatigue. As such, tachycardia > 110/min lasting shorter than 8 min may be considered normal whereas durations longer than 43 min are very abnormal as they only occur in 5% of patients. Alert fatigue is a main barrier for the clinical uptake of CVSM as similar systems in the ICU, PACU and OR results in hundreds of alerts [6] and ultimately alert fatigue where also the true positive alert is ignored [7, 8]. This adds to the discussion on evidence-based alert thresholds to address the alert fatigue issue, where setting an alert at 88% for > 45 min would result in alerting for 50% of patients with no complications. Quantifying this trade-off systematically (modelling alert burden against missed events across thresholds and durations) is a natural next step, but beyond the scope of this descriptive study. Even so, we emphasize that a safe duration for individual patients cannot be defined without regard to the individual patient, as well as careful consideration of complementary data from patients with complications.

### Strengths

Occurrence of AEs and SAEs were evaluated through manual assessment of medical records and laboratory results, in accordance with a standardized outcome manual. Assessments were performed by trained medical experts, and not simple extraction of complication codes, improving the quality of outcomes assessment. Secondly vital sign data was collected continuously via validated wireless equipment ensuring high data-capture except for SBP which was intermittent.

### Limitations

This descriptive study is limited to the description of complication-free patients. It cannot be used to describe what constitutes healthy trajectories, as we would need to include patients with complications. The problem then becomes which complications are relevant and the timing of the complications to interpret. A main limitation related to the blood-pressure measurements, where the available devices only allow for monitoring 30 and 60 min apart, and with relatively reduced data-completion. Thus, we cannot rule out that longer durations of SBP deviations than observed can have occurred in between measurements. Furthermore, due to the discomfort from the cuff-based BP-technique, patients may have been awakened and thus not have long durations of hypotension.

Study participation did not mean that patients were not monitored according to guidelines and manual EWS measurements were still conducted. Our study could not account for treatments that were initiated based on these scores and the clinical observations made by hospital staff during random visits. However, we had blinded the staff to the continuously assessed vital signs and the data should represent the most valid vital sign collection within ethical boundaries.

## Conclusion

Episodes of severe desaturations, tachycardia and tachypnea were short, but moderate desaturations were frequent when complication free patients are monitored continuously. These findings may suggest alert thresholds based upon what is rarely seen in complication free- postoperative patients and should be explored in future trials.

## Supplementary Information

Below is the link to the electronic supplementary material.

Supplementary Material 1

## Data Availability

No datasets were generated or analysed during the current study.
