# Supplementary material for: Vital signs during complication-free recovery after major surgery: a retrospective analysis
Source: J Clin Monit Comput. 2026 Jul 11;40(5):1147–54. doi: 10.1007/s10877-026-01471-7 (PMC13562176; doi:10.1007/s10877-026-01471-7)
Supplement: Supplementary file 1 [file 10877_2026_1471_MOESM1_ESM.docx]

**Supplemental**

**Table 1a: Statistical distribution characteristics of patients without complications.**

|  | Raw values | | | | | Log transformed values | | | | | |
| --- | --- | --- | --- | --- | --- | --- | --- | --- | --- | --- | --- |
|  | **Mean [SD]** | **Median [IQR]** | **Percentiles 5th–95th** | **Skewness / Kurtosis** | **SW test p-value** | **Mean [SD]** | **Median [IQR]** | **Percentiles 5th–95th** | **Skewness / Kurtosis** | **SW test p-value** | **Back-transformed mean (95% CI)** |
| SpO2 < 92 | 89.5 [109.6] | 58.0 [6 – 129] | 0 – 304.2 | 1.97 / 4.82 | 0 | 1.48 [0.82] | 1.77 [0.85 – 2.11] | 0.0 – 2.48 | -0.6 / -0.86 | 0 | 30.4 (24.3 – 37.9) |
| SpO2 < 88 | 27.0 [46.02] | 9.0 [2 – 31.0] | 0 – 114.8 | 3.41 / 16.02 | 0 | 1.00 [0.64] | 1.00 [0.48 – 1.51] | 0.0 – 2.06 | 0.2 / -0.90 | 0 | 10.0 (8.4 – 12.0) |
| SpO2 < 85 | 11.2 [29.04] | 3.0 [1 – 9.0] | 0 – 48.6 | 7.34 / 72.39 | 0 | 0.65 [0.56] | 0.60 [0.30 – 1.00] | 0.0 – 1.70 | 0.7 / -0.13 | 0 | 4.5 (3.9 – 5.2) |
| SpO2 < 80 | 3.3 [10.59] | 1.0 [0 – 3.0] | 0 – 14.0 | 7.35 / 64.03 | 0 | 0.34 [0.40] | 0.30 [0.00 – 0.60] | 0.0 – 1.18 | 1.5 / 2.52 | 0 | 2.2 (1.9 – 2.4) |
| Respiration Rate < 5 | 0.2 [0.72] | 0.0 [0 – 0.0] | 0 – 1.0 | 5.25 / 36.56 | 0 | 0.06 [0.15] | 0.00 [0.00 – 0.00] | 0.0 – 0.30 | 2.7 / 8.07 | 0 | 1.1 (1.1 – 1.2) |
| Respiration Rate < 11 | 15.6 [40.36] | 4.0 [2 – 12.0] | 0 – 67.2 | 7.34 / 68.37 | 0 | 0.83 [0.51] | 0.70 [0.48 – 1.11] | 0.0 – 1.83 | 0.8 / 0.51 | 0 | 6.8 (5.9 – 7.8) |
| Respiration Rate > 24 | 5.6 [13.46] | 1.0 [0 – 4.0] | 0 – 22.8 | 4.40 / 21.17 | 0 | 0.47 [0.47] | 0.30 [0.00 – 0.70] | 0.0 – 1.37 | 1.1 / 0.86 | 0 | 3.0 (2.6 – 3.4) |
| Respiration Rate > 35 | 0.5 [3.31] | 0.0 [0 – 0.0] | 0 – 2.0 | 10.85 / 121.71 | 0 | 0.05 [0.19] | 0.00 [0.00 – 0.00] | 0.0 – 0.48 | 5.1 / 32.28 | 0 | 1.1 (1.1 – 1.2) |
| Heart Rate < 30 | 0.2 [1.22] | 0.0 [0 – 0.0] | 0 – 1.0 | 7.32 / 59.80 | 0 | 0.04 [0.15] | 0.00 [0.00 – 0.00] | 0.0 – 0.30 | 4.9 / 25.21 | 0 | 1.1 (1.0 – 1.1) |
| Heart Rate < 50 | 6.2 [22.91] | 0.0 [0 – 1.0] | 0 – 42.0 | 5.72 / 41.05 | 0 | 0.25 [0.52] | 0.00 [0.00 – 0.30] | 0.0 – 1.63 | 2.3 / 4.38 | 0 | 1.8 (1.5 – 2.0) |
| Heart Rate > 111 | 14.9 [35.20] | 3.0 [0 – 13.0] | 0 – 70.4 | 5.48 / 37.88 | 0 | 0.71 [0.63] | 0.60 [0.00 – 1.15] | 0.0 – 1.85 | 0.5 / -0.68 | 0 | 5.1 (4.3 – 6.1) |
| Heart Rate > 131 | 3.9 [14.29] | 0.0 [0 – 1.0] | 0 – 20.4 | 5.87 / 40.27 | 0 | 0.24 [0.45] | 0.00 [0.00 – 0.30] | 0.0 – 1.33 | 2.1 / 4.03 | 0 | 1.7 (1.5 – 1.9) |
| SBP < 70 | 0.1 [0.87] | 0.0 [0 – 0.0] | 0 – 0.0 | 14.57 / 225.49 | 0 | 0.01 [0.09] | 0.00 [0.00 – 0.00] | 0.0 – 0.00 | 8.8 / 90.57 | 0 | 1.0 (1.0 – 1.1) |
| SBP < 90 | 2.8 [6.55] | 0.0 [0 – 2.1] | 0 – 16.8 | 3.25 / 12.11 | 0 | 0.27 [0.44] | 0.00 [0.00 – 0.49] | 0.0 – 1.25 | 1.4 / 0.74 | 0 | 1.8 (1.6 – 2.1) |
| SBP > 180 | 3.0 [9.61] | 0.0 [0 – 2.1] | 0 – 13.3 | 7.09 / 61.90 | 0 | 0.27 [0.43] | 0.00 [0.00 – 0.49] | 0.0 – 1.15 | 1.6 / 1.74 | 0 | 1.8 (1.6 – 2.1) |
| SBP > 220 | 0.4 [3.13] | 0.0 [0 – 0.0] | 0 – 1.2 | 14.61 / 226.73 | 0 | 0.04 [0.17] | 0.00 [0.00 – 0.00] | 0.0 – 0.34 | 5.8 / 40.44 | 0 | 1.1 (1.0 – 1.1) |

Table 1a. Table describing the raw and log-transformed longest durations of vital sign deviations after major surgery in uncomplicated patients. Systolic blood pressure values are described in percent of all measurements. Logarithmic transformations were performed on all values greater than 0.
SBP: Systolic blood pressure, SW-test = Shapiro-Wilks test for normality, SpO_2_ = peripheral oxygen saturation

**Table 1b: Statistical distribution characteristics of patients with complications.**

|  | Raw values | | | | | Log transformed values | | | | | |
| --- | --- | --- | --- | --- | --- | --- | --- | --- | --- | --- | --- |
|  | **Mean [SD]** | **Median [IQR]** | **Percentiles 5th–95th** | **Skewness / Kurtosis** | **SW test p-value** | **Mean [SD]** | **Median [IQR]** | **Percentiles 5th–95th** | **Skewness / Kurtosis** | **SW test p-value** | **Back-transformed mean (95% CI)** |
| SpO2 < 92 | 74.6 [96.8] | 40.0 [4 – 102] | 0 – 261.4 | 2.19 / 5.9 | 0 | 1.38 [0.83] | 1.61 [0.70 – 2.01] | 0.0 – 2.42 | 0.6 | 0 | 23.8 (19.3 – 29.4) |
| SpO2 < 88 | 33.6 [65.0] | 9.0 [2 – 32.0] | 0 – 157.8 | 3.53 / 14.7 | 0 | 1.02 [0.68] | 1.00 [0.48 – 1.52] | 0.0 – 2.20 | 0.3 / -0.77 | 0 | 10.5 (8.9 – 12.5) |
| SpO2 < 85 | 13.4 [37.2] | 3.0 [1 – 9.0] | 0 – 69.5 | 7.44 / 73.6 | 0 | 0.68 [0.58] | 0.60 [0.30 – 1.00] | 0.0 – 1.85 | 0.8 / 0.05 | 0 | 4.8 (4.1 – 5.5) |
| SpO2 < 80 | 4.1 [17.0] | 1.0 [0 – 2.0] | 0 – 15.9 | 11.21 / 155.4 | 0 | 0.34 [0.42] | 0.30 [0.00 – 0.48] | 0.0 – 1.23 | 1.7 / 3.42 | 0 | 2.2 (2.0 – 2.4) |
| Respiration Rate < 5 | 0.2 [0.69] | 0.0 [0 – 0.0] | 0 – 1.0 | 7.97 / 78.8 | 0 | 0.04 [0.13] | 0.00 [0.00 – 0.00] | 0.0 – 0.30 | 3.7 / 16.82 | 0 | 1.1 (1.1 – 1.1) |
| Respiration Rate < 11 | 9.6 [20.7] | 3.0 [1 – 8.0] | 0 – 41.9 | 4.97 / 30.4 | 0 | 0.69 [0.48] | 0.60 [0.30 – 0.95] | 0.0 – 1.63 | 0.8 / 0.42 | 0 | 4.9 (4.4 – 5.5) |
| Respiration Rate > 24 | 5.1 [13.6] | 1.0 [0 – 4.0] | 0 – 25.9 | 5.94 / 46.1 | 0 | 0.41 [0.48] | 0.30 [0.00 – 0.70] | 0.0 – 1.43 | 1.2 / 0.69 | 0 | 2.6 (2.3 – 2.9) |
| Respiration Rate > 35 | 0.2 [1.22] | 0.0 [0 – 0.0] | 0 – 0.9 | 10.87 / 131 | 0 | 0.03 [0.13] | 0.00 [0.00 – 0.00] | 0.0 – 0.27 | 6.3 / 45.27 | 0 | 1.1 (1.0 – 1.1) |
| Heart Rate < 30 | 0.1 [0.69] | 0.0 [0 – 0.0] | 0 – 1.0 | 8.34 / 82.3 | 0 | 0.03 [0.12] | 0.00 [0.00 – 0.00] | 0.0 – 0.30 | 4.8 / 26.48 | 0 | 1.1 (1.0 – 1.1) |
| Heart Rate < 50 | 3.9 [20.8] | 0.0 [0 – 1.0] | 0 – 9.9 | 9.94 / 123.2 | 0 | 0.19 [0.41] | 0.00 [0.00 – 0.30] | 0.0 – 1.04 | 2.9 / 9.06 | 0 | 1.6 (1.4 – 1.7) |
| Heart Rate > 111 | 24.7 [81.3] | 1.0 [0 – 10.5] | 0 – 141.6 | 5.68 / 38.0 | 0 | 0.60 [0.73] | 0.30 [0.00 – 1.06] | 0.0 – 2.15 | 1.1 / 0.29 | 0 | 4.0 (3.3 – 4.8) |
| Heart Rate > 131 | 3.4 [18.3] | 0.0 [0 – 0.0] | 0 – 14.0 | 9.97 / 115.5 | 0 | 0.18 [0.42] | 0.00 [0.00 – 0.00] | 0.0 – 1.18 | 2.6 / 6.97 | 0 | 1.5 (1.4 – 1.7) |
| SBP < 70 | 0.2 [1.3] | 0.0 [0 – 0.0] | 0 – 0.7 | 7.35 / 58.4 | 0 | 0.03 [0.15] | 0.00 [0.00 – 0.00] | 0.0 – 0.22 | 5.1 / 27.07 | 0 | 1.1 (1.0 – 1.1) |
| SBP < 90 | 5.4 [12.4] | 0.0 [0 – 4.7] | 0 – 29.1 | 3.62 / 14.9 | 0 | 0.38 [0.53] | 0.00 [0.00 – 0.75] | 0.0 – 1.48 | 1.1 / 0.00 | 0 | 2.4 (2.1 – 2.8) |
| SBP > 180 | 1.9 [6.04] | 0.0 [0 – 0.0] | 0 – 10.1 | 5.12 / 30.1 | 0 | 0.19 [0.38] | 0.00 [0.00 – 0.00] | 0.0 – 1.05 | 2.0 / 3.18 | 0 | 1.5 (1.4 – 1.7) |
| SBP > 220 | 0.2 [1.20] | 0.0 [0 – 0.0] | 0 – 1.0 | 10.95 / 144.8 | 0 | 0.03 [0.14] | 0.00 [0.00 – 0.00] | 0.0 – 0.29 | 5.3 / 31.82 | 0 | 1.1 (1.0 – 1.1) |

Table 1b. Table describing the raw and log-transformed longest durations of vital sign deviations after major surgery in complicated patients. Systolic blood pressure values are described in percent of all measurements. Logarithmic transformations were performed on all values greater than 0.
SBP: Systolic blood pressure, SW-test = Shapiro-Wilks test for normality, SpO_2_ = peripheral oxygen saturation

Table 2: Longest desaturation episodes for representative surgery types.

|  | Any complication | | | | No complications | | | | | | |  |
| --- | --- | --- | --- | --- | --- | --- | --- | --- | --- | --- | --- | --- |
|  | n | <80 | <85 | <88 | | <92 | n | <80 | <85 | <88 | <92 | |
| Colorectal and Small Bowel Surgery | 54 | 2.0 [1.0 - 3.0] | 3.0 [2.0 - 9.0] | 12.0 [3.0 - 38.0] | | 59.0 [23.5 - 142.5] | 113 | 2.0 [1.0 - 3.8] | 5.0 [2.0 - 12.0] | 9.0 [4.0 - 31.0] | 71.5 [22.2 - 152.8] | |
| Pancreatic Surgery | 112 | 2.0 [1.0 - 8.8] | 5.5 [2.0 - 16.5] | 14.0 [4.0 - 34.0] | | 59.0 [22.8 - 125.0] | 58 | 3.0 [1.0 - 6.5] | 6.0 [2.0 - 14.0] | 16.0 [4.0 - 42.0] | 62.0 [29.0 - 142.0] | |
| Esophageal Surgery | 65 | 2.0 [1.0 - 3.0] | 6.5 [2.0 - 13.8] | 11.0 [3.0 - 36.0] | | 59.0 [27.5 - 98.0] | 12 | 1.0 [1.0 - 1.5] | 2.5 [1.0 - 11.0] | 10.0 [6.0 - 26.0] | 80.0 [41.0 - 137.0] | |
| Rectal Surgery | 22 | 1.5 [1.0 - 4.5] | 3.0 [1.0 - 9.5] | 5.5 [2.8 - 35.8] | | 29.0 [16.0 - 141.0] | 23 | 3.0 [1.5 - 3.5] | 4.0 [2.5 - 9.0] | 7.0 [3.5 - 17.0] | 47.5 [19.0 - 67.0] | |
| Gastric and Duodenal Surgery | 18 | 5.0 [2.0 - 7.0] | 5.0 [2.5 - 17.8] | 16.5 [5.2 - 38.8] | | 82.0 [25.5 - 184.2] | 24 | 3.0 [2.0 - 5.0] | 8.0 [2.2 - 16.0] | 10.5 [3.2 - 54.0] | 95.0 [38.0 - 185.5] | |
| Abdominal Wall and Peritoneal Surgery | 15 | 3.0 [2.0 - 3.0] | 6.0 [4.0 - 24.5] | 12.5 [4.2 - 57.0] | | 74.0 [38.2 - 157.5] | 15 | 4.0 [3.0 - 10.0] | 7.5 [3.2 - 14.8] | 16.5 [5.2 - 43.8] | 118.0 [42.0 - 175.0] | |
| Biliary Surgery | 4 | 1.0 [1.0 - 1.0] | 6.5 [4.8 - 8.2] | 9.0 [5.0 - 13.5] | | 34.0 [28.0 - 40.0] | 5 | 1.0 [1.0 - 1.0] | 4.5 [1.8 - 15.0] | 27.5 [5.5 - 54.2] | 114.5 [37.5 - 194.0] | |
| Retroperitoneal Surgery | 4 | 3.0 [1.0 - 5.2] | 4.5 [2.5 - 24.8] | 35.0 [11.2 - 127.8] | | 339.0 [240.0 - 396.5] | 4 | 3.5 [3.0 - 9.0] | 19.0 [11.5 - 24.5] | 48.5 [21.2 - 72.8] | 83.0 [72.5 - 101.2] | |
| Popliteal and Distal Arterial Surgery | 4 | 3.0 [2.0 - 4.0] | 4.0 [3.8 - 6.2] | 4.0 [3.8 - 24.2] | | 10.0 [10.0 - 10.0] | 3 | 1.0 [1.0 - 1.0] | 1.0 [1.0 - 1.0] | 1.0 [1.0 - 1.0] | 2.0 [2.0 - 56.0] | |
| Femoral Artery Surgery | 3 | 17.0 [10.5 - 23.5] | 7.0 [4.0 - 20.5] | 14.0 [8.0 - 25.0] | | 29.0 [16.0 - 45.0] | 7 | 1.0 [1.0 - 2.5] | 3.0 [1.0 - 9.0] | 3.0 [2.0 - 12.0] | 3.0 [1.0 - 7.0] | |
| Lymphatic Surgery | 3 | 2.0 [2.0 - 2.0] | 15.0 [10.0 - 24.0] | 162.0 [83.5 - 168.5] | | 59.0 [59.0 - 59.0] | 2 | 1.5 [1.2 - 1.8] | 2.0 [2.0 - 2.0] | 3.0 [2.5 - 3.5] | 15.5 [8.8 - 22.2] | |

Longest period of desaturation for each patient across all monitored days for each patient.
Note: Only procedures with more than 2 procedures in each group is shown for simplicity

**Figure 1: Proportion of patients with a vital sign deviation (any duration)**
